# Absolute quantification of rare gene targets in limited samples using crude lysate and ddPCR

**DOI:** 10.1038/s41598-025-94115-w

**Published:** 2025-03-21

**Authors:** Charandeep Kaur, Stuart Adams, Catherine N. Kibirige, Becca Asquith

**Affiliations:** 1 Department of Infectious Disease, Imperial College London, London, UK; 2 SIHMDS-Haematology, Great Ormond Street Hospital for Children, London, UK

**Keywords:** ddPCR, Crude lysate, Rare targets, Limited sample, Lysate buffer, TRECs, Gene expression analysis, Molecular biology, Biological techniques, Isolation, separation and purification

## Abstract

Accurate quantification of rare genes from limited clinical samples is crucial for research purposes but is technically challenging, especially due to nucleic acid extraction. Using the commercially available genomic DNA (gDNA) extraction kits, which mostly include a DNA purification step through silica columns, magnetic beads or ethanol precipitation, are the preferred choice for many researchers. These kits, however, have a minimum cell number requirement for optimal DNA quality and yield. They are not ideal for use for clinical samples with limited cell numbers. Here, we report the development and validation of a novel crude lysate method for preparing DNA for the absolute quantification of rare genes, TRECs in our case, by droplet digital PCR (ddPCR), from infrequent cells, that removes the need for DNA extraction. Multiple optimization steps and analytical validation of this novel assay was performed on PBMCs extracted from the blood of healthy donors. The newly developed assay shows good agreement with standard ddPCR and has high accuracy, specificity, and reproducibility; additionally, it can also be applied to fixed and permeabilized cells. The assay has the potential to be used for quantification of other trace targets from limited cell samples.

**Supplementary Information:**

The online version contains supplementary material available at 10.1038/s41598-025-94115-w.

## Introduction

Recent technological advances have transformed digital polymerase chain reaction (dPCR) from being an expensive technique with limited application, to a readily-accessible, widely-applied technology^1^. With its exceptional analytical sensitivity and precision over quantitative PCR/ real-time PCR (qPCR/RT-PCR)^2–4^, dPCR can be utilized for analysis of gene expression, epigenetics, copy number variation, rare mutations, linkage studies and quantification of next-generation sequencing libraries^1–8^.

In dPCR, a sample is divided into many small partitions, each of which undergoes PCR amplification. Poisson statistics are used to determine the absolute quantity of a target sequence in a sample based on the proportion of partitions in which the sequence is detected^9,10^. This approach permits absolute quantification without the need for a standard curve, offering an advantage over qPCR^9^.

Despite many advances in dPCR technology, its performance is still problematic when the amount of starting material is limited, for instance, in studies or diagnostic tests involving small subpopulations of human cells. This problem is partially solved by droplet digital PCR (ddPCR); a form of dPCR that uses water-oil emulsion droplets to partition the sample^10^. ddPCR can work with nM to pM concentrations of DNA^10^. However, DNA extraction from ~ < 1000 cells is technically difficult and can lead to target loss during the extraction process. Commercially available kits for DNA extraction work well for cultured cells or for samples with abundant cell quantity (mostly greater than a hundred thousand cells). There is, therefore, a need for a simple, robust and high yield method of preparing DNA from limited number of cells to quantify rare targets using ddPCR.

Here we report a study to quantify T-Cell Receptor Excision Circles (TRECs), a rare target using optimized crude lysate ddPCR. TRECs are small loops of extrachromosomal DNA formed in T cells during recombination of the T cell receptor genes in the thymus. TRECs are not replicated during mitosis and so are diluted in the cell population with each round of cell division^11^. TREC measurement is used to quantify thymic output and cell division history and is implemented in newborn screening programs for Severe Combined Immunodeficiency^12^. Different subpopulations of T cells have different concentrations of TRECs depending on the number of cell divisions they have undergone since thymic production. Consequently, the naïve T cell subpopulation has markedly higher TREC concentrations than memory T cell subpopulations^13^. Quantification of TRECs in low-frequency memory T subpopulations e.g. T stem cell memory cells (which constitute 2–4% of the total CD4 + and CD8 + T cell population in blood)^13,14^, or antigen-specific T cells (in absence of acute infection, one cell in 100 to 10^5^ T cells^15^), are problematic for two reasons: the target is rare and the starting cell numbers low. We therefore sought to develop a simple method for DNA preparation from minimum of 200 cells that is compatible with ddPCR.

## Results

In order to have an assay to compare our novel crude lysate ddPCR assay with we first optimized an in-house standard ddPCR assay (Supplementary File, “optimization and validation of standard ddPCR using extracted DNA”) which we validated against a well-established qPCR method developed at Great Ormond Street Hospital for clinical diagnostics. We found our in-house standard ddPCR assay to be accurate and comparable with the qPCR method (Supplemental Fig. S1). Henceforth, we use this standard ddPCR assay as a benchmark for our novel ddPCR assay (i.e. to test its accuracy), our goal was an assay as accurate as the standard ddPCR assay but able to work with fewer cells and rare targets.

### Optimization and validation of crude lysate DdPCR assay

Different methods were investigated for preparing cell lysates including thermal lysis, sonication, lysis buffer from DNA extraction kit, Ambion Cell to-Ct^®^ kit (“Buffer 1”) and lysis buffer from SuperScript™ IV CellsDirect™ cDNA Synthesis Kit (“Buffer 2”) (Methods). Of the different methods, lysis Buffer 1 and Buffer 2 were the most promising and were taken forward (Supplemental Fig. S2).

### Impact of viscosity breakdown protocol

**Fig. 1:**
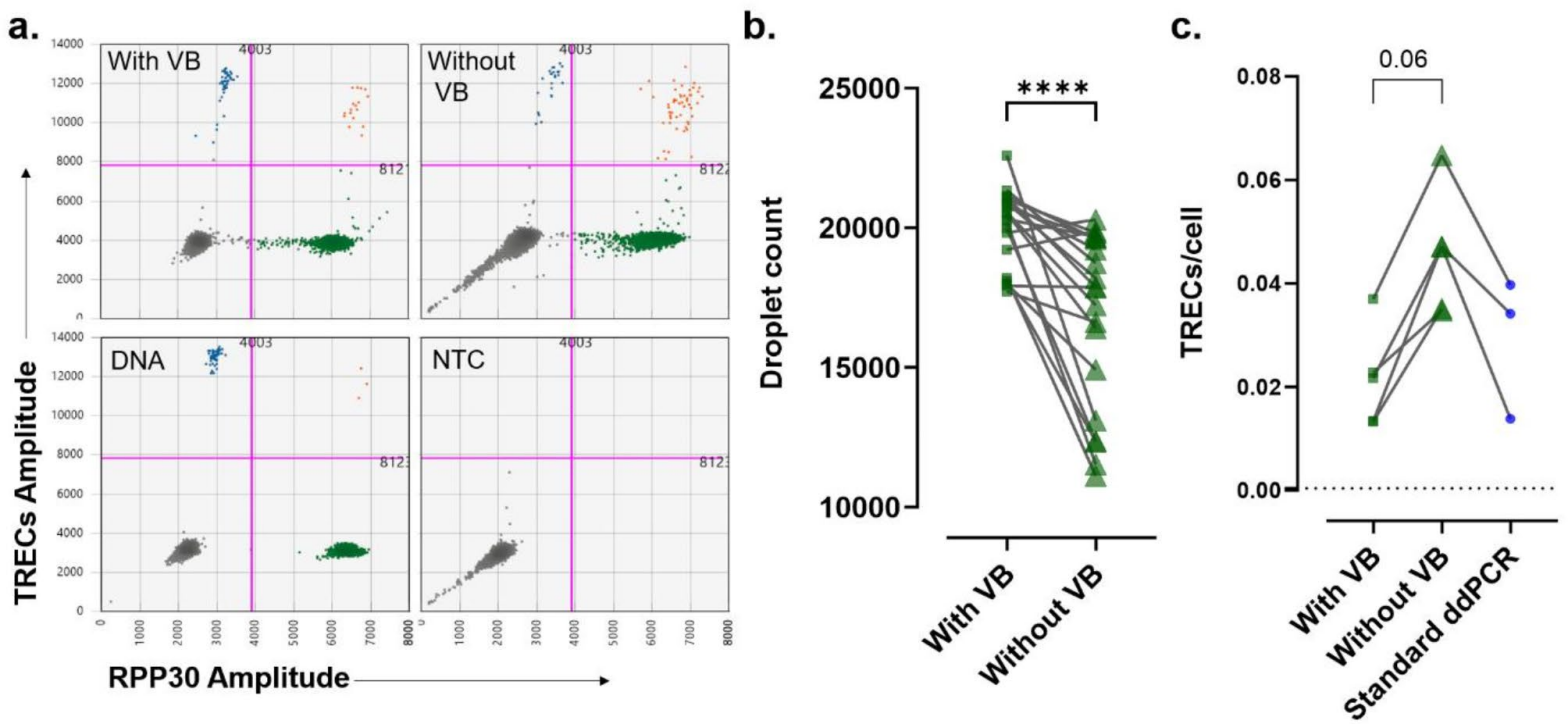
Importance of viscosity breakdown step in crude lysate. (**a**) 2-D dot plots show results of crude lysate ddPCR performed on PBMCs (one representative plot from 5 healthy individuals) processed with and without viscosity breakdown (VB) step. Standard ddPCR and NTC were included as controls. Blue dots are TRECs single positive, green dots are *RPP30* single positive, Orange dots are TREC *RPP30* double positive and grey dots are double negative. The pink line represents the threshold. Each dot represents a droplet in ddPCR. (**b**) Scatter plot shows pairwise comparisons between the total droplet count obtained from crude lysate ddPCR with VB (green squares) and without VB (green triangles). Each symbol represents one droplet. Stats: Wilcoxon test. (**c**) Pairwise comparisons were made between TRECs copies/cell measured by crude lysate ddPCR with VB (green squares) and without VB (green triangles) and the standard ddPCR (blue dots). Each symbol represents a donor, and pairwise comparison is shown by connecting black lines. Stats: Wilcoxon test.

The presence of intact oligonucleotides in crude cellular lysates increases viscosity, posing challenges for droplet formation and target amplification^16^. To reduce this problem, we added an innovative viscosity breakdown (VB) step to lysed cells prior to droplet formation^17^. To assess the impact of this step, we performed crude lysate ddPCR with and without the VB protocol. In most of the experimental runs without VB, we found an unexpected spread of droplets lying on the diagonal in the 2D plot, making it hard to determine the threshold to separate positive and negative cells (Supplemental Fig. S3). In the experiments where this pattern was not observed (Fig. 1a), we found a significant difference between the number of droplets generated between samples processed with and without the VB protocol (Fig. 1b). Moreover, we observed higher levels of TREC copies/cell in the samples processed without the VB protocol (*n* = 5, mean = 0.046 TRECs/cell) compared to standard ddPCR (*n* = 3, mean = 0.029) (Mann Whitney, *p* = 0.1), while no difference was observed between samples processed with VB protocol (*n* = 5, mean 0.022) and standard ddPCR (Mann Whitney, *p* = 0.4) (Fig. 1c). These results suggest that the VB step improves reliability and accuracy.

### Droplet volume

**Fig. 2:**
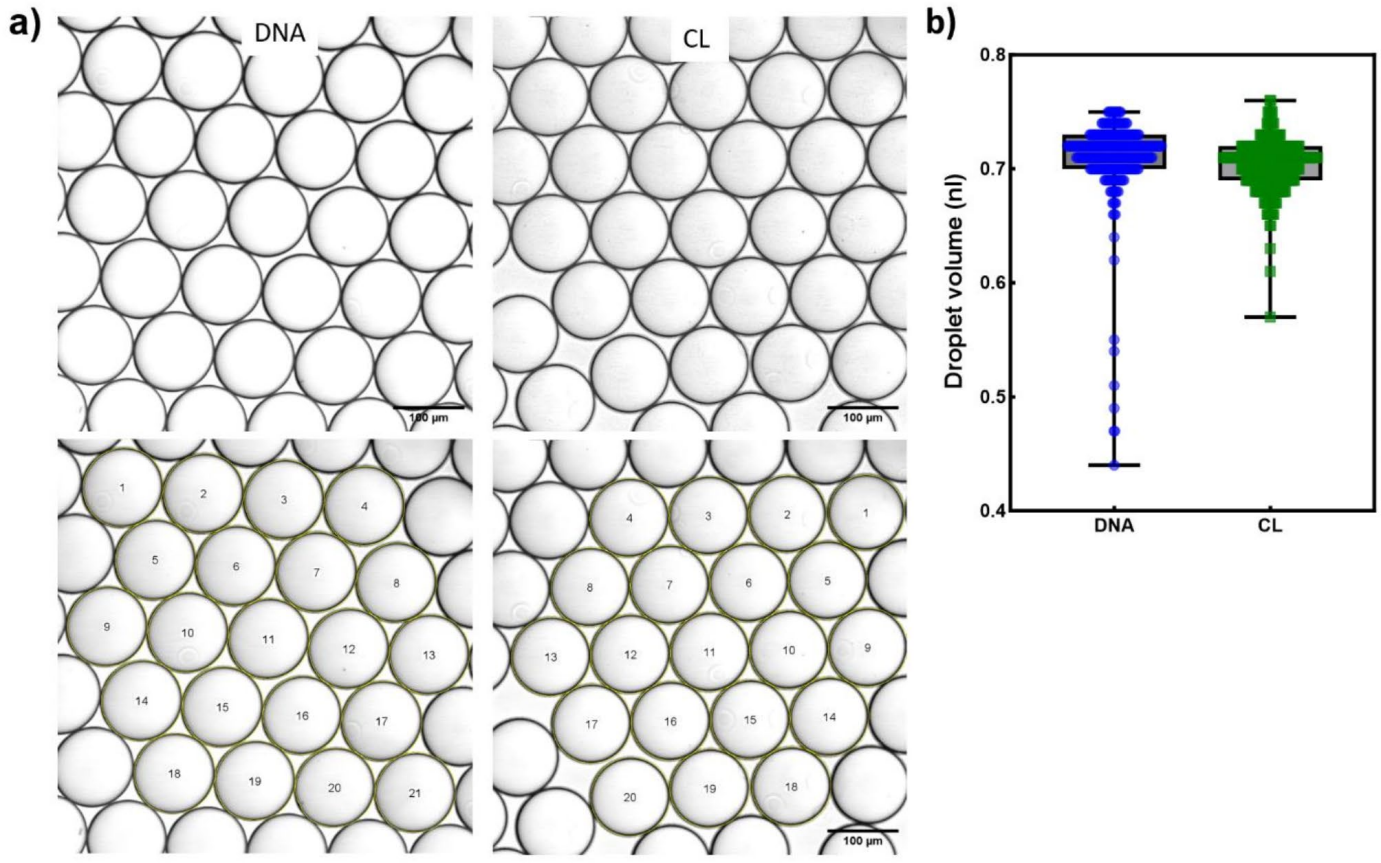
The ddPCR Droplet Volume measurement. Optical microscopy images were used to measure droplet volume. (**a**) A monolayer of droplets was generated using extracted DNA and CL observed under a Leica SP8 Confocal optical microscope. Bottom row shows the images after processing using ImageJ software which involved thresholding and analyzing particle size determination. (**b**) Box-whiskers plot shows the median droplet size of droplets containing extracted DNA (in blue) and CL (in green), each circle represents one droplet.

We were concerned that the cell lysate buffers may affect the droplet volume. We therefore examined, by optical microscopy, droplets generated using crude lysate and compared this with droplets generated using extracted DNA. To prevent any potential alteration in droplet size during microscopic examination, the images were captured within 30 min of droplet generation (Fig. 2a).

The average droplet volume generated using DNA was 0.7096nL (SD 0.037; 95%CI 0.7054–0.7138) and using crude lysate was 0.7034nL (SD 0.022; 95%CI 0.7010–0.7058) (Fig. 2b), both of which are smaller than the value of 0.85nL used in QuantaSoft v1.0.596 to calculate copy number concentration. Therefore, going forward, we used 0.70nL as the droplet volume in our calculations for ddPCR using CL and DNA.

We found no evidence that the crude lysate protocol significantly impacted droplet volume; an unexpected benefit of the crude lysate protocol was that it appeared to reduce variability in the droplet volume.

### Assay linearity and accuracy

**Fig. 3:**
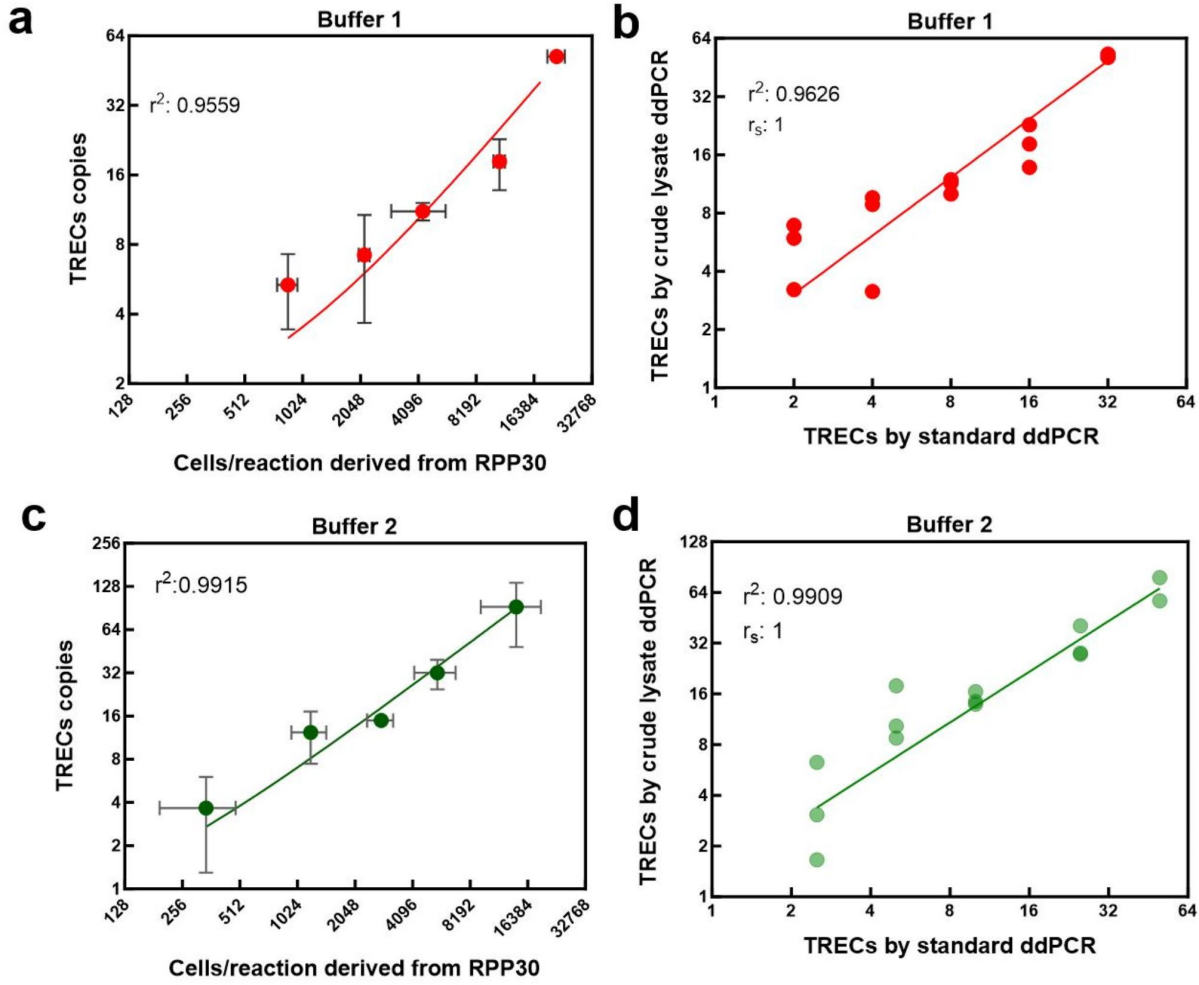
Linearity and accuracy of crude lysate ddPCR using lysis buffer 1 and 2. (**a**–**d**) Linear regression analysis performed on the standard curve made by diluting PBMCs in PBS and then preparing crude lysate using lysis Buffer 1 (in Red, **a**,**b**) or Buffer 2 (in green, **c**,**d**). The PBMC samples used for Buffer 1 and Buffer 2 experiments were distinct, with varying TREC concentrations. (**b**,**d**) The accuracy of the assay was estimated by comparing the TRECs concentration measured by crude lysate ddPCR using either lysis Buffer 1 (in red) or buffer 2 (in green) with TRECs measured using extracted DNA by standard ddPCR. Linear regression equation for Buffer 1 is Y = 1.513X + 0.5430 and for Buffer 2 is Y = 1.298X + 0.6991. Spearman coefficient r_s_ =1 for both assays.

The linearity of the crude lysate ddPCR assay, prepared using Buffer 1 and Buffer 2 was evaluated and compared with the standard ddPCR. For this, we prepared standard curves by serially diluting PBMCs (200–16,000 cells) in PBS, subsequently lysing the diluted cells using either lysis buffer 1 or buffer 2, covering a range of 2–64 TRECs copies/dilution. For the assay with Buffer 1, a strong linear relationship was observed between the number of cells/reaction (estimated from *RPP30*) and TREC copies, with an r² value of 0.95 (*p* < 0.001), (Fig. 3a). However, measuring the accuracy by comparing the TREC copies measured by the standard ddPCR with TREC copies measured by crude lysate ddPCR using Buffer 1 we found a linear regression equation of Y = 1.513X + 0.5430 (Fig. 3b), indicating a systematic overestimation.

Assay with Buffer 2 also demonstrated good linearity between the number of cells/reaction and TREC copies, (r² value > 0.99, *p* < 0.001) (Fig. 3c). Importantly, TRECs were detected in all the triplicates of the lowest dilution made (from approximately 200 cells). Further comparing the TRECs estimated by standard ddPCR and crude ddPCR, the linear regression equation (Y = 1.298X + 0.6991) had a gradient that was not significantly different to 1 (95%CI:0.97–1.35) (Fig. 3d). Consequently Buffer 2 was taken forward.

### Limit of blank, limit of detection and quantification

**Fig. 4:**
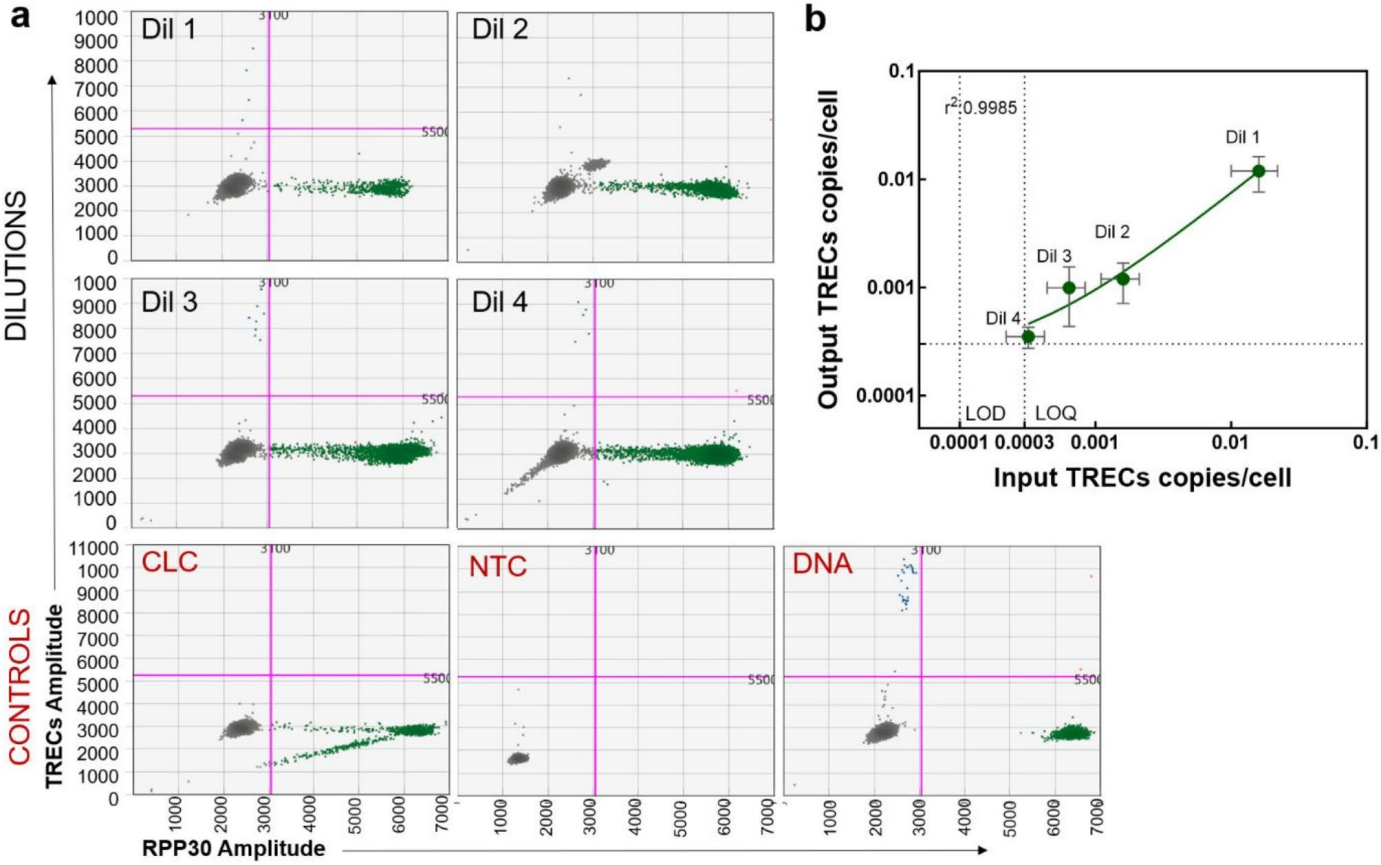
Limit of Detection (LOD) and Limit of Quantification (LOQ) of crude lysate ddPCR using Buffer 2. (**a**) 2-D dot plots depict the results obtained from standard curve samples made by diluting TRECs in monocyte cell line (U937) at concentrations. Additional controls, including CLCs, NTCs and DNA controls, are shown in the bottom three plots (labeled in red). The dot plots represent the merged droplets from triplicate dilutions. Blue dots are TRECs single positive, green dots are *RPP30* single positive, orange dots are TREC *RPP30* double positive and grey dots are double negative. The pink line represents the threshold. Each dot represents a droplet in ddPCR. (**b**) Linear regression analysis between the average TRECs copies/cell measured by standard ddPCR (Dil1: 0.016 ± 0.006 TRECs/cell, Dil2: 0.0016 ± 0.0005 TRECs/cell, Dil3: 0.0006 ± 0.0002 TRECs/cell and Dil4: 0.0003 ± 0.0001 TRECs/cell) and the TRECs copies/cell measure by crude lysate ddPCR. Each dot represents the mean and the error bars indicate the standard deviation. Linear regression between TRECs estimated by standard ddPCR vs. TRECs estimated by ddPCR from cell lysate showed a good linearity down to 0.0003 TRECs/cells, r^2^ >0.99, *p* < 0.001 with good agreement between the two methods (Y = 0.7286*X + 0.0002274).

Limit of Blank (LOB) for crude lysate ddPCR assay was estimated to be zero (Supplemental Fig. S4). To estimate the Limit of Detection (LOD) of crude lysate ddPCR using Buffer 2, TREC copies were quantified in samples of four different cell concentrations made by diluting PBMCs (of known TRECs/cell, estimated by standard ddPCR), in the monocyte cell line. The total cell numbers or TREC concentrations of the dilutions made for lysis ranged from 200 − 10,000 cells or 2 to 64 TREC copies/cell. TRECs were successfully detected in all the dilutions (Fig. 4a, b). However, in Dil 2, an unusual cluster of double-negative appeared slightly higher than the typical double negatives and were excluded manually (Fig. 4a).

Since TRECs were detected in all the samples, we selected the lowest dilution, Dil4 (made by lysing 200 PBMCs within 10,000 U93 cells), to estimate the LOD, and calculated it to be 0.0001 TRECs/cell (LOD: 0 + 1.645*0.00007) (Fig. 4b). The robustness of detection across all dilutions was reaffirmed as a 100% hit rate by probit analysis at all the concentrations. Therefore, we considered Dil4 with an average of 0.0003 TRECs/cell (95% CI 0.0002–0.0005; coefficient of variation (CV) 22%) as the Limit of Quantification (LOQ, Fig. 4b).

### Intra-assay repeatability

We measured CV% of TREC copies/reaction across 8 samples ran in triplicates and observed a mean of 25% CV, ranging from 12 to 40% (Table 1).

**Table 1:** Intra-assay variability (Crude lysate DdPCR using buffer 2). Rep: replicate.

	TREC copies/reaction				
Sample Id	Rep1	Rep2	Rep3	mean	CV%
LD8	20.6694	18.8806	8.8278	16.1259	39.5843
TD1	14.9900	11.7225	13.7664	13.4930	12.2348
TD2	33.1795	46.4623	33.7355	37.7924	19.8809
DPG4	109.7046	78.9029	114.8296	101.1457	19.2124
Dil 1	2.14933	3.510723	3.704297	3.1214	27.1485
Dil 2	3.687285	2.287295	1.714177	2.5629	39.6041
Dil 3	3.718888	6.004963	6.049773	5.2579	25.3523
Dil 4	4.626611	3.227553	3.434326	3.7628	20.0691
Mean					25.38

### Direct comparison with standard DdPCR

**Fig. 5:**
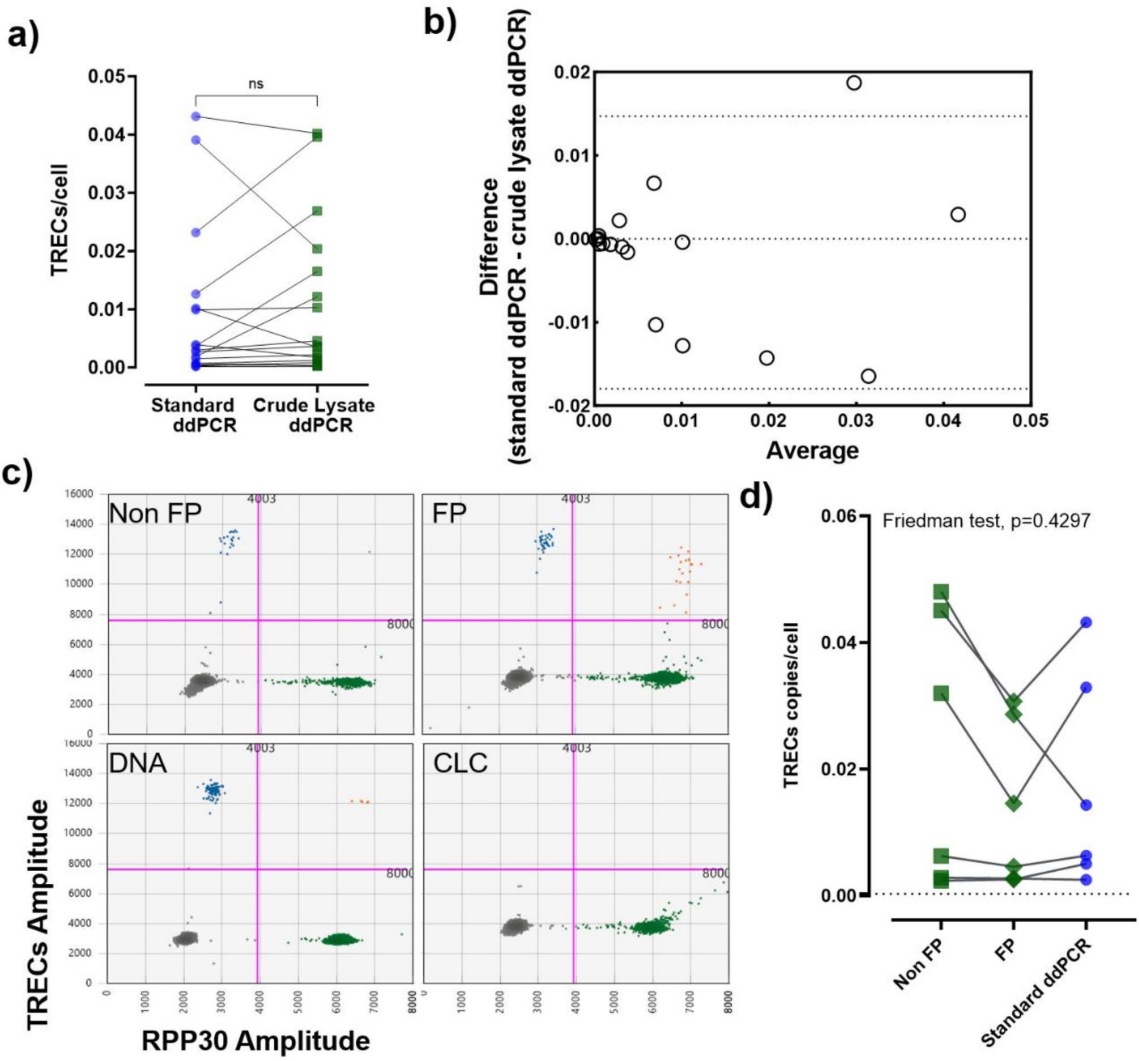
Accuracy of crude lysate ddPCR on fixed and permeabilized (FP) cells. (**a**) A pairwise comparison between TRECs copies/cell detected by the standard ddPCR (in blue circles) and crude lysate ddPCR: FP (in green diamonds) and non FP (in green squares) is plotted. Each symbol represents a donor, and pairwise comparison is shown by connecting black lines. Stats: Wilcoxon test, ns = non significant (*p* > 0.05). (**b**) The Bland-Altman analysis is plotted as a scatter diagram with the TREC copies/cell differences between the two methods on the y-axis and the averages of these two measurements on the x-axis. Horizontal lines are drawn at the mean and at the limits of agreement. (**c**) The 2-D dot plots display TRECs and/or *RPP30* positive droplets detected from paired FP, non FP and extracted DNA samples for a representative sample. Additionally, CLC, a negative control, is also shown. (**d**) A pairwise comparison of TRECs copies/cells obtained from FP (in green diamonds), non FP (in green squares) and extracted DNA (in blue circles) samples. Each symbol represents a donor, and pairwise comparison is shown by connecting black lines. Stats: Friedman test with Dunn’s multiple comparisons, denoting significance as *<0.05. FP: Fixed and permeabilized, non FP: not fixed and permeabilized.

A direct comparison of the newly developed crude lysate ddPCR and the standard ddPCR was made by analyzing TRECs copies/cells using genuine samples (rather than T cells diluted with macrophages). The samples used were sorted T cell subpopulations for which the two assays were run simultaneously. Mean TREC copies/cell were not significantly different between the two assays (Wilcoxon test, *p* = 0.31) (Fig. 5a). The Bland-Altman analysis indicated a bias of -0.001635 between two methods with SD of 0.008337. The small negative bias indicates that, on average, standard ddPCR tends to slightly underestimate the TREC copies compared to ddPCR directly with cell lysate (Fig. 5b) whilst the small standard deviation suggests the methods are relatively consistent.

### Impact of cell fixation and permeabilization

Extracting DNA from fixed and permeabilized cells is challenging due to the crosslinking of proteins and nucleic acids. However, it is often necessary to work with fixed, permeabilized samples. We therefore investigated the impact of fixation and permeabilization on TRECs quantification using crude lysate ddPCR with buffer 2. Six different samples of fixed and permeabilized cells were compared with paired non-fixed cells and with standard ddPCR (Fig. 5c). The TREC count was not significantly different between these three groups (*p* = 0.4) (fixed: mean = 0.014, non-fixed: mean = 0.022, standard ddPCR: mean = 0.017). However, the TREC count tended to be lower in fixed cells compared to non-fixed cells for the three samples with a higher TREC concentration (Fig. 5d).

## Discussion

Absolute quantification of rare genes from limited clinical samples is challenging yet crucial for research and clinical purposes. Most traditional and commercial nucleic acid extraction methods require an optimal number of cells to perform within desirable specifications. These methods can incur significant target loss which may be problematic when dealing with limited cell numbers. To address this an assay based on crude lysate was developed and optimized.

The accuracy of this novel protocol was compared to standard ddPCR. Overall, the novel assay showed good agreement with the standard assay in the range from 0.0003 to 0.01 TRECs/cell; whilst being simultaneously more sensitive.

Quantifying the performance of the new assay we found the LOB to be zero, the LOD to be < 0.0001 TREC/cell and the LOQ to be < 0.0003 TRECs/cell. This is favourable compared to standard ddPCR for which we found the LOB to be zero, the LOD to be 0.0004 TRECs/ cell and the LOQ to be 0.002 TRECs/cell. This relatively high LOQ for standard ddPCR is consistent with previous reports. Profaizer & Slev (2020) determined the LOQ for TRECs using standard ddPCR to be 2 copies/µl of blood^18^ and for qRT-PCR to be 24 copies/µl of blood^18^; which (using an estimate of 1*10^3^ − 2*10^3^ PBMCs per µl of blood) is equivalent to a LOQ of 0.002 − 0.001 TRECs/cell for standard ddPCR and 0.024 − 0.012 TRECs/cell for qRT-PCR, approximately 3 and 40 times higher, respectively, than our novel assay. We found that the novel assay worked with as few as 200 cells.

Unexpectedly, we found that the droplet volume obtained using crude lysate (0.7nL) was lower than that assumed by the QuantaSoft software v1.0.569 (0.85nL). This decrease in volume from that expected was not attributable to the crude lysate protocol as we found an almost identical droplet volume with the standard ddPCR assay (0.7nl). Whilst some studies have found a droplet volume similar to Bio-Rad’s estimates^19^, our observation is consistent with a number of previous studies using the Biorad system that have shown deviations from the default droplet volume^20,21^. Notably, Koˇsir et al. (2017) reported a droplet volume of (0.71nL, very) similar to our findings^22^.

It has been noticed that standard ddPCR underestimated TREC copies slightly when compared to crude lysate ddPCR (Fig. 5b) and had higher variability in droplet volume (Fig. 2b) suggesting that the use of crude lysates instead of kit-extracted DNA actually improves the ability to detect TRECs.

Quantifying repeatability we find an average CV of 25% (Table 1). This variability may arise from the viscosity of the PCR mix, incomplete mixing of samples or the presence of secondary DNA structures which might disturb the random distribution of samples. Additionally, variations in sample handling, pipetting or minor inconsistencies in assay conditions could also impact variability.

Techniques for rare samples often involve a pre-amplification reaction. Pre-amplification increases the copy number of nucleotide sequences in the reaction before PCR, enabling the analysis of genes with limited nucleic acid content. In pre-amplification reactions, there are two major challenges: augmenting the reaction’s capacity and ensuring target amplification specificity^23^. Failing to tackle these challenges can introduce significant bias. By circumventing the need for pre-amplification, we eliminated related issues. Simply put, fewer steps in the process reduce the potential for introducing error.

Our method differs from previously published studies on crude lysate ddPCR for absolute quantification of genes^24–26^, as we utilized the whole cell lysate instead of a portion of it. Working with a larger volume of cell lysate presents challenges as it alters the viscosity of the reaction mixture, resulting in a reduction in droplet count and experiment efficiency. We successfully addressed this challenge by introducing a viscosity breakdown step. This approach is more robust for detecting rare events from limited clinical or biological samples. By partitioning a single sample of cell lysates into four ddPCR reactions (to fully consume the lysate), we were able to comprehensively screen the entire sample to detect rare genes. Subsequently, these four samples were merged, this significantly increased the number of screened droplets per sample.

Furthermore, we conducted experiments to evaluate the efficacy of this protocol on fixed cells. Although there were no significant differences observed between fixed/permeabilized cells and non-fixed cells, it was noted that the count of TRECs tended to be lower in the fixed cells compared to the non-fixed cells. We suggest that further optimization is needed before this method can be used on fixed and permeabilized samples.

Plasmids are unsuitable for optimizing PCR as they can lead to overestimation of the target^27^. Therefore, for the assay optimisation, we used PBMCs rather than plasmids containing TRECs DNA. In adherence to MIQE guidelines, we consistently included negative and positive controls to assess background signals and set the threshold^28^.

The study’s limitations include a relatively small number of replicates used for optimization and technical assessment. Furthermore, while our LOD and LOQ assessment indicated the presence of TRECs copies in all dilutions, we did not explore cell concentrations below 200 cells and TREC concentration below 0.0003 TRECs copies/cell due to the satisfactory performance within the optimized range. We encountered occasional artifacts, such as double negative clusters or isolated droplets with significantly higher TRECs and *RPP30* amplitudes than the rest. These artifacts may be attributed to contamination, debris in the crude lysate, or technical variations, such as temperature fluctuations or minor inconsistencies during droplet generation.

The optimised high-throughput crude lysate ddPCR assay holds immense promise across various fields of molecular biology. This advancement has wide-ranging implications, particularly in quantifying rare events such as circulating tumor cells, fetal DNA in maternal plasma, rare genetic mutations, and the analysis of precious clinical samples where cell and gene target quantity is limited but precision is crucial.

In summary, the novel protocol of crude lysate ddPCR is accurate, sensitive, and precise in estimating rare gene from limited samples. By eliminating the DNA extraction step and utilizing the whole cell lysate, the protocol minimizes potential sources of variability, such as DNA loss, and provides a robust approach for the accurate analysis of rare gene events.

## Methods

### Peripheral blood samples and monocyte cell line

Peripheral blood (60-70 ml) from healthy donors were collected in Sodium Heparin tubes (BD Vacutainer) and processed within 3 h. PBMCs were isolated via density gradient centrifugation over Histopaque^®^Hybri-max (Sigma-Aldrich). The PBMC layer was collected, washed with PBS, and cryopreserved in 10% Dimethyl Sulfoxide (Merck) in Fetal Calf Serum (FCS) (heat-inactivated, Gibco).

When required, cryopreserved PBMCs were revived in RPMI-1640 media (Sigma) supplemented with 10% FCS. Cells were counted by acridine orange/propidium iodide staining and analyzed using Luna dual Fluorescence cell counter (Logos Biosystems, South Korea). A fraction of these cells was used to prepare standard curve dilutions while the remainder were used for DNA extraction. A monocyte cell line (U-937)/macrophage DNA was used as a negative control.

### Sorting of CD8^+^ T memory subpopulations

For the direct comparison of the newly developed crude lysate ddPCR and standard ddPCR, CD8 + T memory samples (rather than PBMCs or T cells diluted with macrophages) were used. For this, PBMCs were isolated from the blood of healthy volunteer and enriched for CD3^+^ T cells using EasySep™ human T-cell isolation kit (Stemcell Technologies, Vancouver, Canada) as per manufacturer’s instructions. Enriched CD3^+^ T cells were stained with the following fluorochrome-attached antibodies: CD8 (BV711, Biolegend), CD45RA (ECD, Beckman Coulter), CD27 (Qdot605, ThermoFisher), CCR7 (FITC, BD BioScience) and CD95 (Pe-Cy5, Biolegend) antibodies. Stained cells were sorted into different memory subpopulations using a BD FACSAria III-U (BD Biosciences).

### Cell fixation and permeabilization

To investigate the impact of fixation and permeabilization, 2*10^6^ PBMCs were fixed using 200ul of fixation buffer (Invitrogen) for 30 min at room temperature. Fixed cells were washed with 200ul of 1% permeabilization buffer (Invitrogen) and then permeabilized in 200ul of 1% permeabilization buffer for 20 min.

### DNA extraction

Genomic DNA from PBMCs and sorted memory CD8 + T cell subpopulations was extracted using DNeasy Blood and Tissue Kit (Qiagen) as per manufacturer’s instructions. DNA was eluted in 60ul of nuclease free water (NFW) and stored at − 20 °C. DNA concentration was measured fluorometrically by Qubit 3.0 (Invitrogen).

### Cell lysate Preparation

Five different approaches to preparing the cell lysate were compared:

1. Thermal lysis in NFW. 1000-10,000 cells were resuspended in 10ul of NFW and boiled for 5 min at 99 °C on water bath. 10ul of ProteinaseK (10 mg/ml) was added and cells were heated at 57 °C for 30 min, followed by 99 °C for 30 min. Next, cells were rapidly cooled on ice and centrifuged for 2 min at full speed.
2. Sonication. 1000-10,000 cells were resuspended in 50 µl of NFW and sonicated for 1 cycle of 30 s with Peak Power = 105, Duty factor = 10 and cycle/burst = 200.
3. Lysis buffer from DNeasy Blood & Tissue Kit (Qiagen). Only lysis reagents were used as per manufacturer’s instructions.
4. Lysis in Buffer (1) Lysis reagents from the Ambion Cell to-Ct^®^ kit (ThermoFisher) were used. Cell lysate was prepared according to manufacturer’s instructions, omitting the DNase DNA degradation step.
5. Lysis in Buffer (2) Lysis reagents from the SuperScript™ IV CellsDirect™ cDNA Synthesis Kit (ThermoFisher) were used. Cell lysate was prepared according to manufacturer’s instructions, omitting the DNase DNA degradation step.

Cell lysate prepared using the methods above was used in the ddPCR reaction mixture.

### Cell lysate viscosity breakdown step

The cell lysate was heated on a thermocycler at 65 °C for 1 min, 96 °C for 2 min, 65 °C for 4 min, 96 °C for 1 min, 65 °C for 1 min and 96 °C for 30 s. Tubes were spun for 1 min and cooled at room temperature before using in ddPCR reaction mixture^17^.

### DdPCR reaction mixture Preparation

All the preparation steps and reaction set up were performed in a dedicated pre-PCR room and in a dedicated PCR hood. Two probes were used: a TRECs probe labelled with FAM and a housekeeping gene *RPP30* (ribonuclease P/MRP subunit p30) probe labelled with HEX. Primers and probe used for TRECs: Forward primer 5’-CAC ATC CCT TTC AAC CAT GCT-3’; Reverse primer 5’-GCC AGC TGC AGG GTT TAG G-3’, Probe Sequence: 5’-ACA CCT CTG GTT TTT GTA AAG GTG CCC ACT-3’ with FAM and BQ-1 quencher^29^. For *RPP30*, we used the primer probe mix for copy number detection from BioRad.

A 22ul reaction mixture was prepared comprising 11ul 2×ddPCR Supermix for probes (no dUTP) (Bio-Rad, CA, USA), 0.55 ul of TRECs primers (36 μm stock) and probes (10 μm) each, 1.1 ul of *RPP30* copy number detection (Bio-Rad), 1ul of HindIII-HF restriction enzyme (NEB, UK). The remaining 7.8ul was adjusted with NFW and extracted DNA for standard ddPCR. Or, for crude lysate ddPCR, 7.8ul of cell lysate was used. For buffer 1, seven replicates were made and for buffer 2, four replicates were made. During data analysis, these replicates were summed so that a total of ~ 140,000 droplets/sample were screened for buffer 1 and 80,000 droplets/sample for buffer 2.

### DdPCR droplet generation and thermocycler protocol

From the 22ul of ddPCR reaction mixture, 20 µl was loaded into a droplet DG8 cartridge (Bio-Rad, USA, #1864008) in sample loading wells. 70ul of droplet oil was loaded in the droplet oil wells. The DG8 cartridge was covered with a gasket and placed in the droplet generator (Bio-Rad #186–3002). 40ul of droplets generated in the cartridge were transferred to a 96-well plate. The plate was sealed and put on the thermocycler (Bio-Rad, USA) under the following conditions: 10 min hold at 95 °C, 45 cycles of 94 °C for 30s then 59 °C for 60s, next step of 98 °C for 10 min and final hold at 12 °C. Amplified droplets were then read in the QX200 Droplet Reader (Bio-Rad, USA). The droplet reader was turned on for 30 min before reading the plate.

### Droplet volume size estimated by optical microscopy

Droplet volume was compared between standard and crude lysate ddPCR. Droplet generation and optical microscopy were performed on the same day. Four wells were randomly selected for both ddPCR methods from different cartridges. 80–100 droplets/well were measured to get approximately 300 droplets in total. The droplets were transferred to µ-Slide VI flat uncoated microscopic chamber (IBIDI Germany). The slide was held at an angle for a few seconds to obtain a uniform monolayer of droplets. An optical microscope (Leica SP8 confocal microscope) with a digital camera was used to image the droplets. Images were recorded under uniform illumination in a bright field imaging mode, with 200x magnification. The 2-D images were analyzed in ImageJ (2.9.0) following an existing procedure^20^.

### Assay linearity

The linearity and accuracy of the standard ddPCR and crude lysate ddPCR were assessed separately. For standard ddPCR assay, a standard curve of five serial dilutions (in triplicates) was made using CD3 + T cell DNA diluted with macrophage DNA (macrophages are negative for TRECs).

For crude lysate ddPCR using buffer 1 and buffer 2, 200 − 10,000 cells were used to make two-fold serial dilutions in PBS covering a range of 2–64 TRECs copies/dilution. Serially diluted cells were then lysed using either buffer 1 or buffer 2 (“Cell lysate preparation”, methods). The housekeeping gene *RPP30* served as an internal control for quantifying cell numbers.

### Limit of blank

Limit of Blank (LOB) was calculated using the TRECs copy number concentration in a total of 22 non-template controls (NTCs) and 22 U-937 monocyte cell line lysate controls (CLCs) reactions using the formula^30^:

$${\text{LOB}}={\text{Mea}}{{\text{n}}_{({\text{copy}}\;{\text{number}}\;{\text{blank}})}}+1.645{\text{ }} \times {\text{ }}S{D_{({\text{copy}}\;{\text{number}}\;{\text{blank}})}}.$$

### Limit of detection, and limit of quantification

Standard ddPCR: A standard curve was made by diluting DNA isolated from PBMC with DNA concentration ranging from 3ng/ul-0.001ng/ul.

Crude lysate ddPCR: A standard curve made from PBMCs (200–10,000 cells) diluted with the U-937 monocyte cell line was used. A total of 4 dilutions were made with TRECs concentration ranging from 0.01 to 0.0003 TRECs/cell, run in triplicates.

The Limit of Detection (LOD) and Limit of Quantification (LOQ) were calculated as previously described (18). Specifically, the LOD was the lowest copy number concentration that could be distinguished from the LOB with 95% certainty, calculated by equation^30^:

$${\text{LOD}}={\text{LOB}}+1.645 \times {\text{S}}{{\text{D}}_{({\text{the}}\;{\text{lowest}}\;{\text{TRECs}}\;{\text{copy}}\;{\text{number}}\;{\text{dilution}})}},$$

While the LOQ was defined as the lowest TRECs copies that could be detected with a coefficient of variation (CV) ≤ 35% ^30^.

### Repeatability of the DdPCR assay

To assess the repeatability (intra-assay variation) of the crude lysate ddPCR assay, TREC copies/cell were measured from 8 samples ran in triplicate. CV% was calculated.

### Data analysis

The ddPCR data were analysed in QuantaSoft version v1.0.596 (Bio-Rad). In each experiment, 3–8 NTCs and/or CLCs reactions were used to define the threshold that differentiates positive droplets from negative droplets. Reactions with > 10,000 accepted droplets/well were used for the analysis. The copy number concentration (copies/ul) was calculated using the formula:

$${\text{Concentration}}= - \ln {\text{ }}\left( {\left( {{{\text{N}}_{({\text{neg}})}}/{\text{N}}} \right)/{{\text{V}}_{{\text{droplet}}}}} \right),$$

where N_(neg)_ is number of negative droplets, N is the total number of droplets and V_droplet_ is the volume of a droplet. We determined V_droplet_ to be 0.70ul by optical microscopy. For crude lysate ddPCR, TRECs/cell were calculated by adding the replicates, seven in case of Buffer 1 and four for Buffer 2. The number of replicates depended on the total volume of the lysate. All statistics were performed in GraphPad Prism version 9.4.1.

### Preprint

This manuscript is uploaded to the preprint server bioRxiv. The DOI is 10.1101/2024.03.27.586936 and the manuscript can be found at https://www.biorxiv.org/content/biorxiv/early/2024/03/28/2024.03.27.586936.full.pdf.

## Electronic supplementary material

Below is the link to the electronic supplementary material.

Supplementary Material 1

Supplementary Material 2

## Data Availability

The datasets generated and/or analyzed during the current study are available in the supplementary file named “Raw_Data” file.

## Abbreviations

TRECs: T-cell receptor excision circles
ddPCR: Droplet digital polymerase chain reaction
dPCR: digital Polymerase Chain Reaction
RT-PCR: Real time - polymerase chain reaction
qPCR: quantitative PCR
PBMC: Peripheral blood mononuclear cells
NFW: Nuclease free water
LOB: Limit of blank
LOD: Limit of detection
LOQ: Limit of Quantification
NTCs: Non-template controls
CLCs: Cell line lysate controls
VB: Viscosity breakdown
PBS: Phosphate buffer saline
Buffer 1: Lysis reagents from the Ambion Cell to-Ct^®^ kit
Buffer 2: Lysis reagents from the SuperScript™ IV CellsDirect™ cDNA Synthesis Kit
Human Genes: Ribonuclease P/MRP subunit p30 (RPP30)
