# Supplementary material for: Absolute quantification of rare gene targets in limited samples using crude lysate and ddPCR
Source: Sci Rep. 2025 Mar 21;15:9744. doi: 10.1038/s41598-025-94115-w (PMC11928631; doi:10.1038/s41598-025-94115-w)
Supplement: Supplementary file 1 — Supplementary Material 1 [file 41598_2025_94115_MOESM1_ESM.docx]

**Supplementary File**

**OPTIMIZATION AND VALIDATION OF STANDARD ddPCR USING EXTRACTED DNA**

In order to have an assay to compare the novel crude lysate ddPCR assay we first optimized and validated an in-house standard ddPCR assay to quantify TREC copies using extracted DNA from PBMCs. Annealing temperatures for TRECs and RPP30 primers and probes were optimized by performing a thermal gradient of 58°C - 62°C degrees. An optimum annealing temperature for both TRECs and RPP30 was determined to be 59°C as this provides the best separation between positive and negative droplets with minimum rain in between them, for both the channels (**Supplemental Figure 1a**).


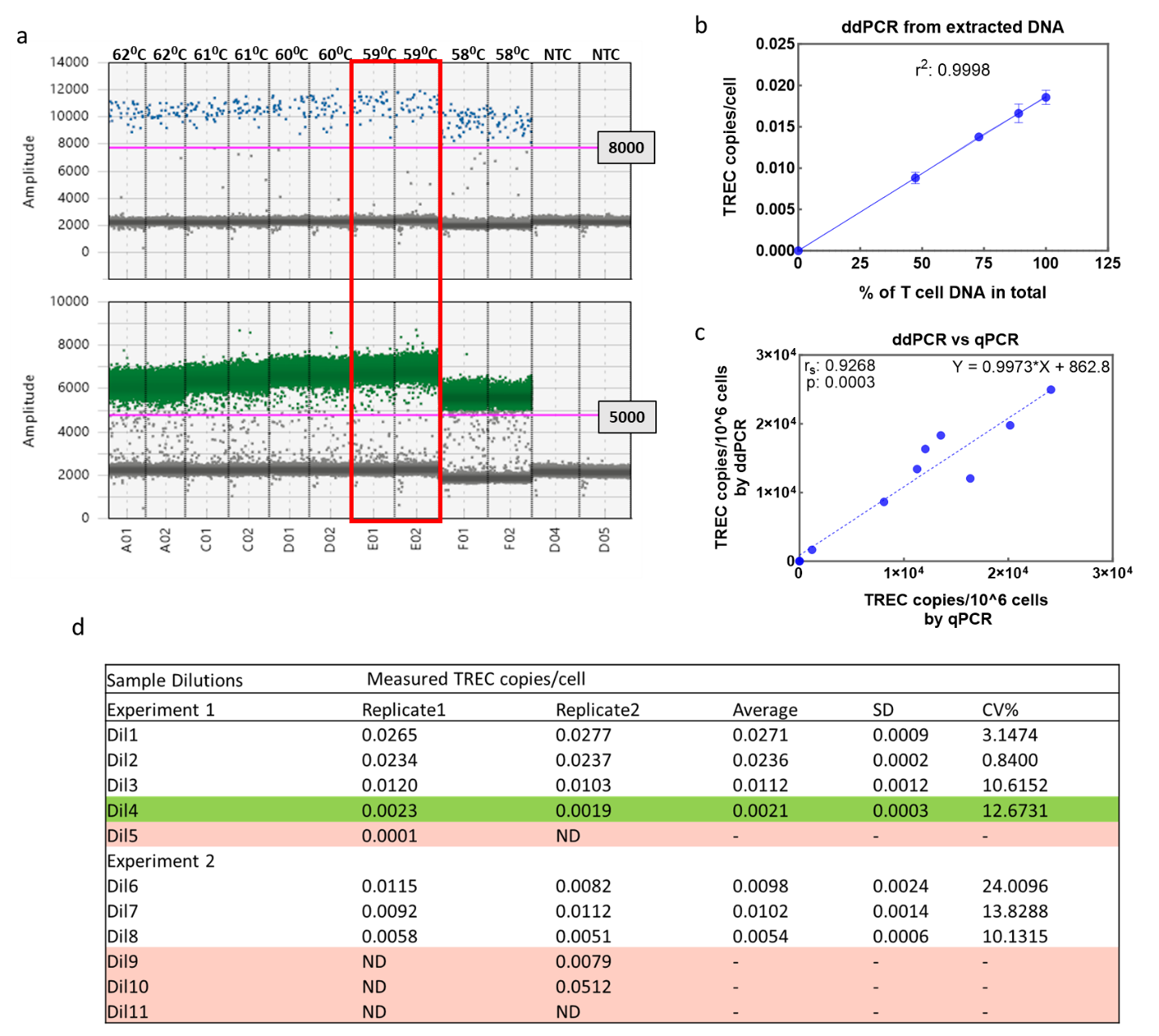


**Supplemental Figure 1: Optimization and validation of standard ddPCR**. (a) Gradient temperature experiment was performed on DNA samples isolated from the blood of a healthy individual and run in duplicates. The upper panel shows a 1-D dot plot displaying the results for the amplitude of TRECs-positive droplets (blue), while the bottom panel presents the results for RPP30, a housekeeping gene(green). The pink horizontal line represents the threshold for positive droplets, and the red squares mark the optimal temperature selected based on the best separation and minimum rain. (b) A standard curve was made by diluting T-cell DNA with macrophage DNA to assess the linearity of the assay. Samples were run in duplicate, the coefficient of determination r_2_ calculated by linear regression. (c) To measure accuracy, TREC copies/million cells were measured in n=10 DNA samples using both qPCR and ddPCR. (d) Dilution experiments for LOD and LOQ estimation. Green row shows the lowest dilution with TRECs detected in both the replicates. Pink rows highlight the dilutions with ND (not detected) in one or both the replicates. SD: Standard Deviation; CV: Coefficient of variation

Next, the linearity of this assay was investigated using serial dilutions of TRECs containing T cell DNA with TREC negative macrophage DNA. TREC copies/cell measured by ddPCR was plotted against percentage of T cell DNA in the reaction mixture and a straight line fitted by linear regression (**Supplemental Figure 1b**). The best fit straight line had a coefficient of determination (r^2^) of 0.99, indicating good linearity between the TRECs copies/cell and the concentration of T cell DNA present in the sample.

Accuracy was assessed by comparing TREC copies/cell quantified from diluted PBMC samples by the standard ddPCR assay with the qPCR method. The qPCR method was implemented by Dr Stuart Adams, Great Ormond St Hospital, London for clinical diagnostics. WE exchanged samples and independently quantified TREC copies/cell. We observed a Spearman coefficient (r_s_) 0.93, with p: 0.0003 between the results obtained by the standard ddPCR and the qPCR assays (n=10) (**Supplemental Figure 1c**). We found the equation of the straight line to be Y = 0.9973*X + 862.8, indicating excellent agreement between the methods over the range studied.

LOB for this assay is zero since we did not detect a single droplet for TRECs in NTCs (Fig S1). TREC copies/cell were detected in the dilutions having ≥ 0.002 TREC copies/cell, however below this concentration we did not detected TRECs in either one or both the replicates (highlighted in pink in **Supplemental Figure 1d**). Hence, we selected the Dil4 with 0.002 TRECs/cell (highlighted in green in **Supplemental Figure 1d**) to estimate the LOD, calculated to be 0.0004 TRECs/cell (LOD: 0+1.645*0.0003). Further, this is the lowest dilution having 100% hit rate for probit analysis and CV%<35, Therefore, we estimate the LOQ to be 0.002 TRECs/cell (coefficient of variation (CV) 12.7%).

These findings confirmed that TREC copies measured by the standard ddPCR method is accurate and comparable with the well-established qPCR method. Henceforth, we use this standard ddPCR assay as a benchmark for our novel crude lysate ddPCR assay.

**CELL LYSATE PREPARATION**

Various methods were tried to make the cell lysates compatible with ddPCR. Initially, we tried a thermal lysis method, droplets were formed and TREC copies/cell were quantified. In the results, the recovery rate of cells, especially for 2000 input cells, was unsatisfactory (Figure S1 a). Given our interest in quantifying TRECs from rare memory subpopulations, a maximum input of 2,000 cells was desired. Subsequently, we explored the sonication method, which only yielded a recovery rate of 11.12% for 2000 cells (Figure S1 b, blue bar). Additionally, we experimented with the lysis buffers from DNA/RNA extraction kits (Qiagen), but these buffers, containing detergent or other harsh chemicals, hindered the droplet formation (data not presented).


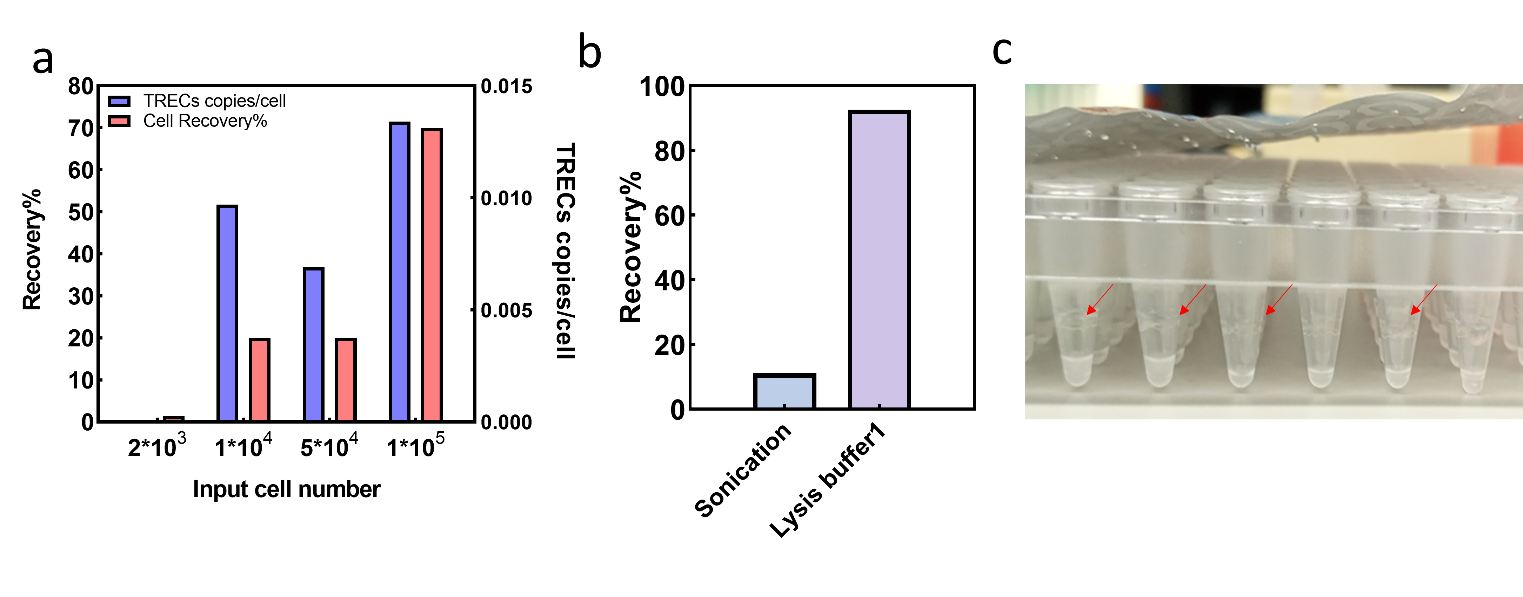


**Supplemental Figure 2: Evaluation of different methods for preparing the crude lysate.** (a) Graph summarizing the results obtained using thermal lysis for making cell lysates. The x-axis represents the input cell number used to make the cell lysates, while the left y-axis shows Recover% calculated as (output cell number estimated from RPP30 copies/input cell number)*100 (Pink bars). The right y-axis represents TRECs copies/cell (Violet bars). (b) Column plot illustrating the Recovery% achieved by lysing 2000 input cells using sonication (blue column) and lysis buffer 1 (purple column) with viscosity breakdown step for preparing cell lysates. (c) Visualization of a 96-well plate after running samples in the ddPCR droplet reader, displaying droplets sticking to the walls of the wells (red arrows).

Our pursuit led us to test lysis reagents from the Ambion Cell to-Ct®kit (ThermoFisher) (Buffer 1), which showed improvements but still faced recovery rate challenges and high standard deviations among replicates (data not showed). To solve this issue, we employed several strategies:

1) Addition of additional DNA polymerase and/or restriction enzyme to the reaction mixture to enhance the amplification efficiency

2) Tried different cell lysate volumes in experiments to make the cell lysates in an attempt to enhance the cell lysis

3) Increased the droplet oil volume from 70ul to 80ul to deal with viscosity

Out of these strategies, only the third was proven effective, suggesting that the viscosity of the crude lysate was impeding target gene amplification. The high viscosity of the reaction mixture caused droplets to adhere to the well’s walls, reducing droplet counts to less than 10,000 (Figure S1 c). Simply increasing the droplet oil volume was insufficient, as its mechanism for addressing viscosity was unclear. Instead, we implemented a viscosity breakdown step and as a result, we achieved over 90% recovery for lysate prepared using buffer1 (Figure S1 b, purple bar).

**VISCOSITY BREAKDOWN PROTOCOL:**


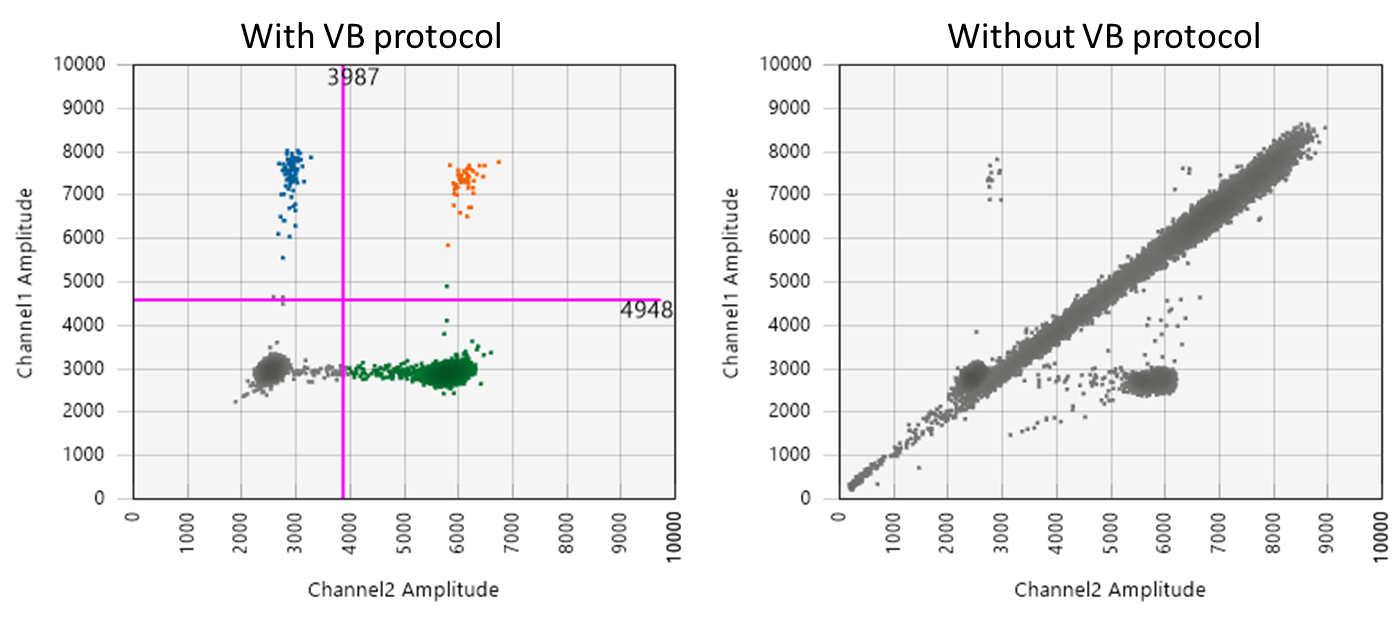


**Supplemental Figure 3. 2-D dot plots showing the effect of viscosity on the droplet distribution performed on PBMCs lysate (representative plot of 4 merged replicated) with and without Viscosity Breakdown (VP) protocol**. Samples processed with VB protocol have less rain between the positive and negative cluster and can be gated manually (Left). While samples processed without VB protocol have lots of droplets run diagonally making it hard to gate the positive and negative cells (Right). Blue dots are TRECs single positive, green dots are RPP30 single positive, Orange dots are TREC RPP30 double positive and grey dots are double negative. The pink line represents the threshold. Each dot represents a droplet in ddPCR.

**
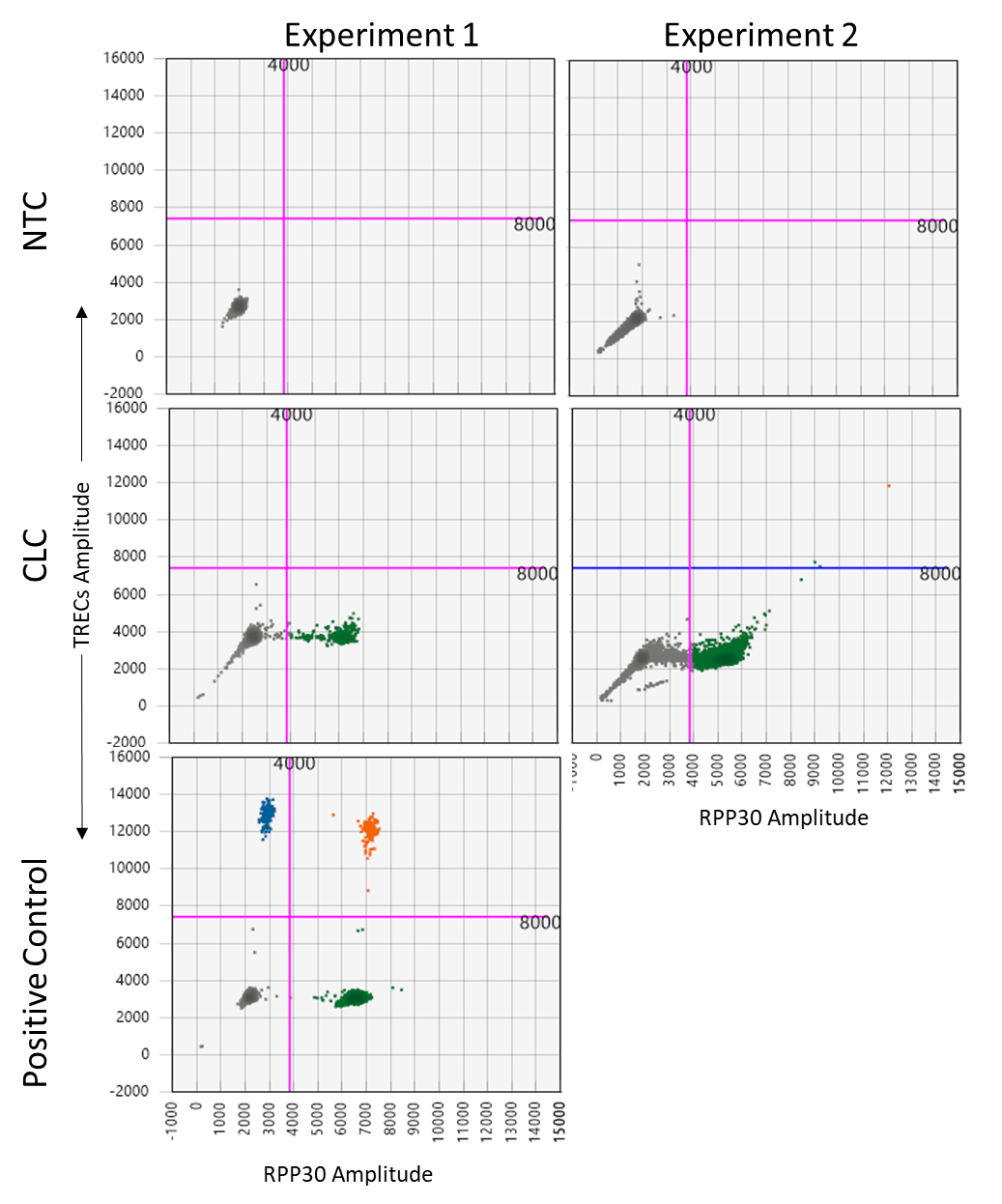
LIMIT OF BLANK**

Non template controls (NTCs) and cell line controls (CLCs) were used to estimate the LOB. None of the NTCs gave a TREC signal or a RPP30 signal (Fig 4a). Likewise, all the CLCs were negative for TRECs (Fig 4a) except for a few very high fluorescent positive droplets lying on the diagonal in a 2D plot which were artifacts and discarded in the analysis. The LOB for the crude lysate assay with buffer 2 was therefore zero.

**Supplemental Figure 4: Limit of Blank (LOB)**. (a) LOB: 2-D dot plots illustrate the results obtained from two separate LOB experiments performed on different days. The dot plots represent the merged droplets from all the samples in one run, in total, there were 22 samples for NTCs (top row) and CLCs (bottom row), 11 samples/run. In addition, a DNA sample isolated from PBMCs was run in triplicate by the standard ddPCR used as a positive control.
